# Supplementary material for: Oceanic crust recycling controlled by weakening at slab edges
Source: Nat Commun. 2020 Apr 24;11:2009. doi: 10.1038/s41467-020-15750-7 (PMC7181835; doi:10.1038/s41467-020-15750-7)
Supplement: Supplementary file 1 — Supplementary Information [file 41467_2020_15750_MOESM1_ESM.pdf]

Oceanic crust recycling controlled by weakening at the slab edges

Munch et al.

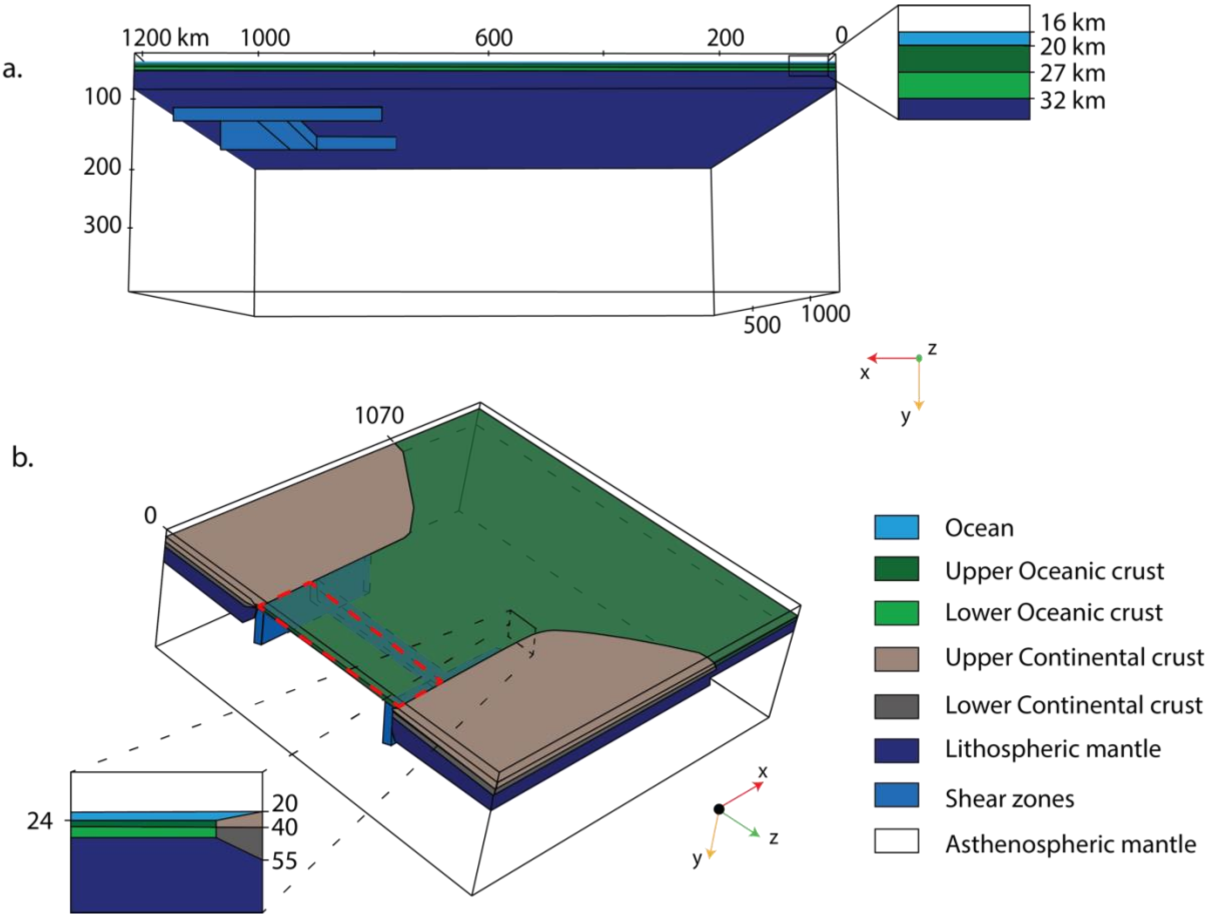

Supplementary *Fig. 1. Initial settings for the oceanic (a.) and continental/oceanic (b.) simulations.* *All lengths are given in kilometers. Oceanic crust is homogeneous around the young lithospheric window and covers the whole box surface (dark and light green layers) for the oceanic simulation a. For the continental/oceanic domain simulation b., the oceanic crust is surrounded by two diverging continental margins (in dark and light grey). "H" shaped weak zones (in light blue) are prescribed in the lithosphere. They enclose the lithospheric young window. The lithosphere thickness is determined by the 1273 K isotherm.*

Supplementary Table 1. Physical properties of rocks used in numerical experiments

Material	Initial density ρ_0 [kg.m^{-3}]	Thermal conductivity K [W.m^{-1}.K^{-1} at T$_K$][36]	Friction coefficient	Flow law[1]
Continental upper crust	2750 (solid)<br>2400 (molten)	1.18+474/(T+77)	0.15	wet quartzite:<br>A_D=1.97x10^{17}, n=2.3,<br>E=154 KJ/mol,<br>C=0.3 MPa, V=0 cm^3,<br>σ_{cr}=3x10^4 Pa
Continental lower crust	2950 (solid)<br>2400 (molten)	1.18+474/(T+77)	0.15	wet quartzite:<br>A_D=4.80x10^{22}, n=3.2,<br>E=238 KJ/mol,<br>C=0.3 MPa, V=0 cm^3,<br>σ_{cr}=3x10^4 Pa
Oceanic upper crust	3000 (solid)<br>2900 (molten)	1.18+474/(T+77)	0.00	wet quartzite:<br>A_D=1.97x10^{17}, n=2.3,<br>E=154 KJ/mol,<br>C=0.3 MPa, V=0 cm^3,<br>σ_{cr}=3x10^4 Pa
Oceanic lower crust	3000 (solid)<br>2900 (molten)	1.18+474/(T+77)	0.10-<br>0.00	plagioclase An$_{75}$:<br>A_D=4.80x10^{22}, n=3.2,<br>E=238 KJ/mol,<br>C=0.3 MPa, V=0 cm^3,<br>σ_{cr}=3x10^4 Pa
Newly formed crust	3000 (solid)<br>2900 (molten)	1.18+474/(T+77)	0.20-<br>0.00	plagioclase An$_{75}$:<br>A_D=4.80x10^{22}, n=3.2,<br>E=238 KJ/mol,<br>C=0.3 MPa, V=0 cm^3,<br>σ_{cr}=3x10^4 Pa
Lithospheric mantle	3300 (solid)<br>2900 (molten)	0.73+1293/(T+77)	0.20-<br>0.00	dry olivine:<br>A_D=3.98x10^{16}, n=3.5,<br>E=532 KJ/mol,<br>C=0.3 MPa, V=8-12 cm^3,<br>σ_{cr}=3x10^4 Pa
Asthenosphere	3300 (solid)<br>2900 (molten)	0.73+1293/(T+77)	0.20-<br>0.00	dry olivine:<br>A_D=3.98x10^{16}, n=3.5,<br>E=532 KJ/mol,<br>C=0.3 MPa, V=8-12 cm^3,<br>σ_{cr}=3x10^4 Pa

Supplementary Table 2. Conditions and results of numerical experiments. Flow laws are taken from[1], thermal conductivity data are taken from [2].

Name	SIW	Mantle activation volume (cm^3)	Initial slab width (km)	Number of continents	Result
swa	Yes	8	480	0	Narrowing of the slab during the retreat.
nswa	No	8	480	0	Widening of the slab during the retreat.
swb	Yes	8	460	0	Narrowing of the slab during the retreat. The slab reaches the boundary of the box before breaking off.
splnswb	Yes	8	660	0	Narrowing of the slab during the retreat.
spsnswb	Yes	8	260	0	Narrowing of the slab during the retreat. Slab breaking off after ~375 km of retreat.
1swc	Yes	8	460	1	Narrowing of the slab during the retreat. The slab reaches the boundary of the box before breaking off. The retreat does not follow the continental margins.
1nswc	No	8	460	1	Widening of the slab during the retreat. The slab retreat does not follow the continental margin.
2swc	Yes	8	460	2	Narrowing of the slab during the retreat. The slab reaches the boundary of the box before breaking off. The retreat does not follow the continental margins.
2nswc	No	8	460	2	Widening of the slab during the retreat. The retreat does not follow the continental margins.
vswb	Yes	12	460	0	Narrowing of the slab during the retreat. The slab stops and start detaching after ~400 km of retreat.
vnswb	No	12	460	0	Widening of the slab during the retreat. The slab stops retreating after ~460 km of retreat.
v2swc	Yes	12	460	2	Narrowing of the slab during the retreat. The slab breaks off after ~535 km of retreat. The retreat does not follow the continental margins
v2nswc	No	12	460	2	Widening of the slab during the retreat. The slab breaks off after 415 km of retreat. The retreat does not follow the continental margins.
vswbat	No	12	460	0	Narrowing of the slab during the retreat. The slab detaches after 280 km of retreat.

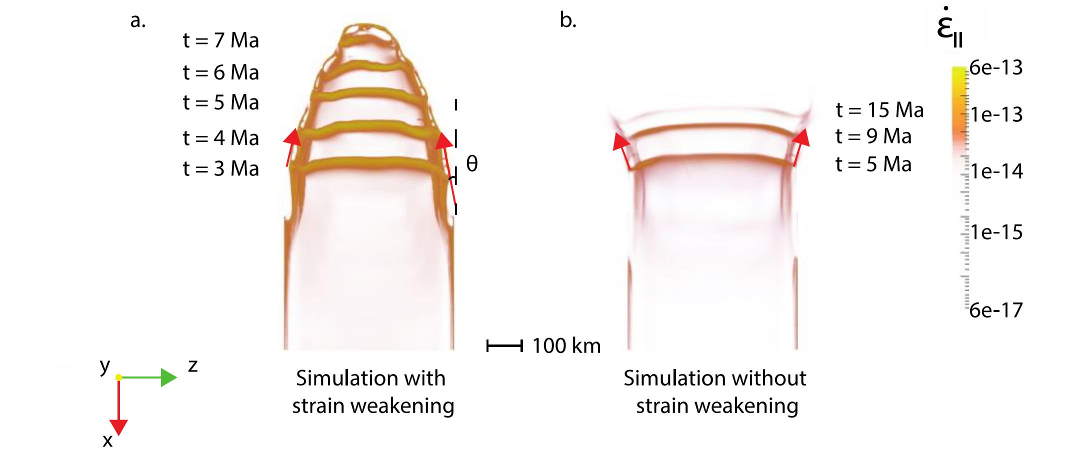

***Supplementary Figure 2. Horizontal deformation at different times for the oceanic-continental models with and without* SIW.**
Models *with SIW are a., at 3, 4, 5, 6 and 7 Ma (Supplementary Figure 1.a., model v2swc in Supplementary Table 2) and the ones without SIW are b., at 5, 9 and 15 Ma (Fig. 4b.,d., model v2nswc in Supplementary Table 2). Note that the slab retreats faster in the simulation with SIW (a.) compared to the one without (b.). Similarly to fully oceanic models (Fig. 2), the angle θ at which the STEP-faults propagate (i.e. the angle of deviation from the slab retreat direction) increases in absolute value with time. In models with SIW, θ increases in the inward direction and tearing paths become more converging with time (a.) whereas in models without SIW, θ increases in the outward direction and tearing paths become more diverging with time (b.).*

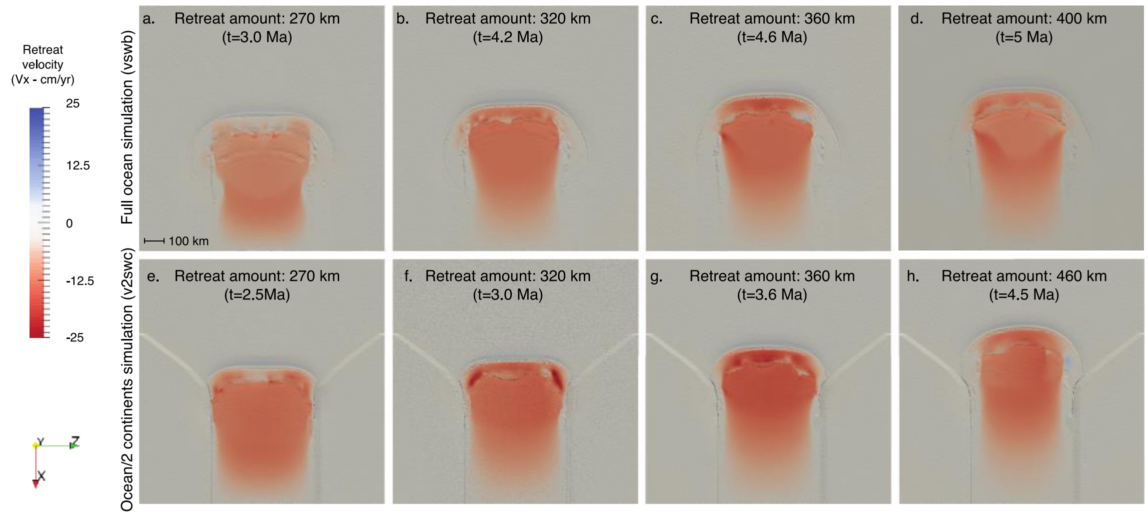

***Supplementary Figure 3: Retreat velocity for simulations with SIW** in 1. fully oceanic domain (simulation vswb, upper line a.-d.) and 2. Mixed ocean-continental setting (simulation v2swc, lower line e.-h.). The retreat occurs in the direction opposite to the axis "x". The retreat appears to have an overall slower rate in the vswb simulation than in the v2swc simulation. The presence of the continents surrounding the retreating slab appears to influence the slab retreat rate (v2swc e.-h.): the slab retreat gets faster when leaving the continental area and slows down once in the oceanic domain.*

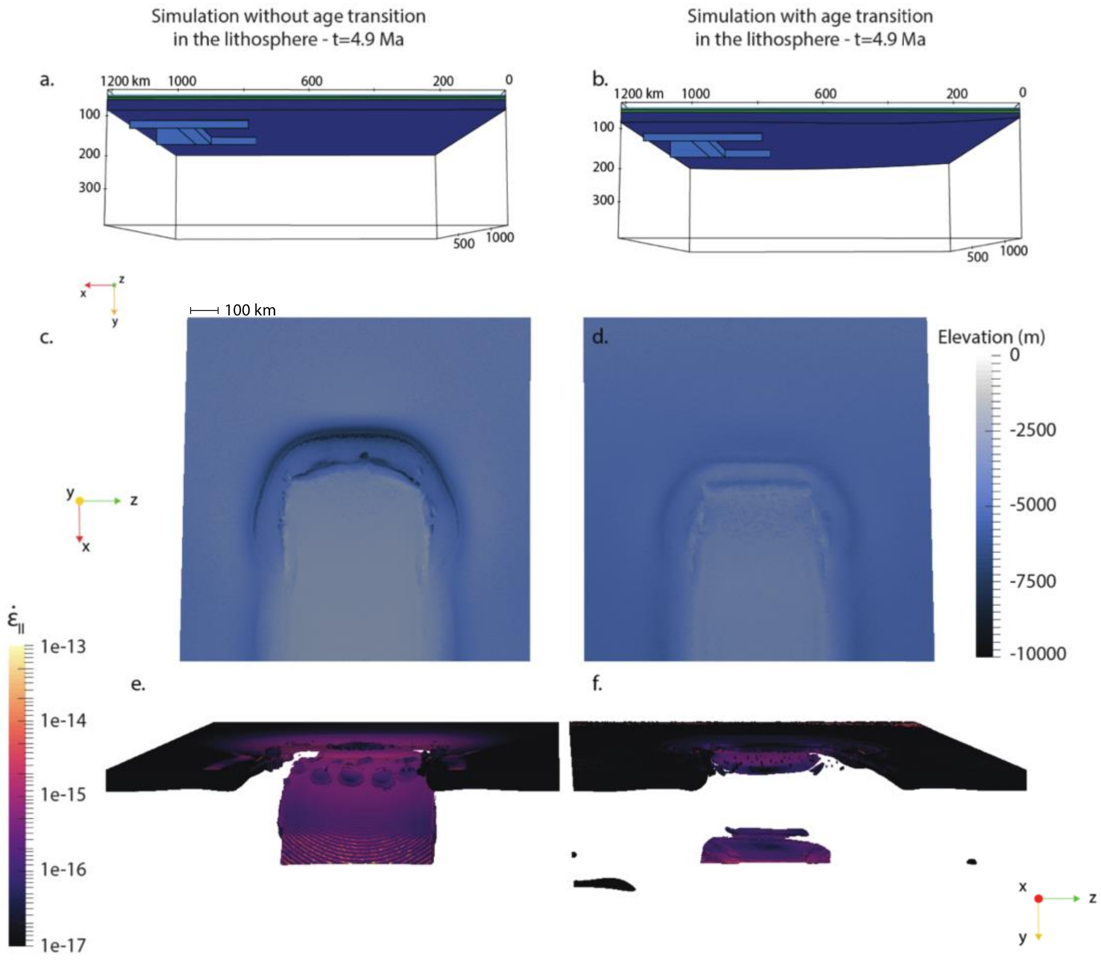

***Supplementary Figure 4: Effect of initial lithosphere age structure**. Initial setting composition field (a. b.), topography (c. d.) and slab shape (e. f.) at 4.9 Ma for two simulations in full oceanic domain with strain weakening. Figs. a. c. and e. correspond to simulation vswb where the lithosphere is initially 40 Ma old everywhere except in the young window. Figs. b. d. and f. show the effect of an initial age variations in the oceanic lithosphere that mimic the effect of lithosphere cooling away from a MOR (simulation vswbat). Age is set to decrease linearly along the x-axis , from 40 Ma at the future position of the subduction initiation , to 10 Ma at the back boundary of the box, assuming a MOR outside of the model domain. During the simulation, the crust gets thicker with time following a half space cooling model. In the case of uniform lithosphere, the slab narrows over time while retreating. When the initial plate age decreases away from the trench (i.e.. slab retreats towards MOR), the evolution is very similar, but the slab breaks off earlier as the younger, thinner lithosphere is both mechanically weaker and more buoyant. Retreat ceases; situation shown slightly later for visual clarity of advanced slab detachment.*

Supplementary references:

1 Ranalli, G. *Rheology of the Earth*. (Springer Science & Business Media, 1995).
2 Ahrens, T. J. *Rock physics & phase relations: A handbook of physical constants*. (1995).
