# Supplementary material for: Oceanic crust recycling controlled by weakening at slab edges
Source: Nat Commun. 2020 Apr 24;11:2009. doi: 10.1038/s41467-020-15750-7 (PMC7181835; doi:10.1038/s41467-020-15750-7)
Supplement: Supplementary file 3 — Description of Additional Supplementary Files [file 41467_2020_15750_MOESM3_ESM.pdf]

Description of Additional Supplementary Files

File Name: Supplementary Movie 1

Description: **Slab retreat in the ocean through time with SIW.** First column: top and side view of the oceanic crust (green) and lithospheric mantle (blue) motion: from subduction initiation to slab retreat (upper panel: top view; lower panel: side view). Central column: Second invariant of the strain rate (amount of deformation in the model), top view in the upper panel, at 60 km depth; 3D high deformation envelope [$\dot{\varepsilon}_{II}$>1.10^{-15}] side view in the lower panel. STEP faults are highlighted by the converging warm colour segments at the edges of the sinking slab. Last column: topography on top of the subduction system. The trench is getting increasingly curved through time as the slab narrows.
